# *Cassava brown streak virus* Ham1 protein hydrolyses mutagenic nucleotides and is a necrosis determinant

**DOI:** 10.1111/mpp.12813

**Published:** 2019-06-01

**Authors:** Katie R. Tomlinson, José Luis Pablo‐Rodriguez, Hamidun Bunawan, Sarah Nanyiti, Patrick Green, Josie Miller, Titus Alicai, Susan E. Seal, Andy M. Bailey, Gary D. Foster

**Affiliations:** ^1^ School of Biological Sciences University of Bristol Life Sciences Building, 24 Tyndall Avenue Bristol BS8 1TQ UK; ^2^ CINVESTAV Campus Irapuato Mexico; ^3^ Institute of Systems Biology (INBIOSIS) Universiti Kebangsaan Malaysia, UKM Bangi 43600 Selangor Darul Ehsan Malaysia; ^4^ National Crops Resources Research Institute (NaCRRI) P.O. Box 7084 Kampala Uganda; ^5^ Natural Resources Institute Chatham Maritime, Kent ME4 4TB UK

**Keywords:** cassava, cassava brown streak disease, cassava brown streak virus, food security, Ugandan cassava brown streak virus, virus

## Abstract

Cassava brown streak disease (CBSD) is a leading cause of cassava losses in East and Central Africa, and is currently having a severe impact on food security. The disease is caused by two viruses within the *Potyviridae* family: *Cassava brown streak virus* (CBSV) and *Ugandan cassava brown streak virus* (UCBSV), which both encode atypical Ham1 proteins with highly conserved inosine triphosphate (ITP) pyrophosphohydrolase (ITPase) domains. ITPase proteins are widely encoded by plant, animal, and archaea. They selectively hydrolyse mutagenic nucleotide triphosphates to prevent their incorporation into nucleic acid and thereby function to reduce mutation rates. It has previously been hypothesized that U/CBSVs encode Ham1 proteins with ITPase activity to reduce viral mutation rates during infection. In this study, we investigate the potential roles of U/CBSV Ham1 proteins. We show that both CBSV and UCBSV Ham1 proteins have ITPase activities through *in vitro* enzyme assays. Deep‐sequencing experiments found no evidence of the U/CBSV Ham1 proteins providing mutagenic protection during infections of *Nicotiana* hosts. Manipulations of the CBSV_Tanza infectious clone were performed, including a Ham1 deletion, ITPase point mutations, and UCBSV Ham1 chimera. Unlike severely necrotic wild‐type CBSV_Tanza infections, infections of *Nicotiana benthamiana* with the manipulated CBSV infectious clones do not develop necrosis, indicating that that the CBSV Ham1 is a necrosis determinant. We propose that the presence of U/CBSV Ham1 proteins with highly conserved ITPase motifs indicates that they serve highly selectable functions during infections of cassava and may represent a euphorbia host adaptation that could be targeted in antiviral strategies.

## Introduction

Cassava (*Manihot esculenta* Crantz, family *Euphorbiaceae*) is the second most important food crop in terms of *per capita* calories consumed in Africa (Nweke, 2004). It can withstand unpredictable rainfall and grows with minimal inputs on marginal land, so provides vital adaptation opportunities to projected climate change (Jarvis *et al.*, 2012). Unfortunately, cassava production in sub‐Saharan Africa is currently limited by two viral diseases: cassava mosaic disease and cassava brown streak disease (CBSD), which have severe impacts on food and economic security across the entire region (Patil *et al.*, 2015; Tomlinson *et al.*, 2017). CBSD is caused by at least two viral species: *Cassava brown streak virus* (CBSV) and *Ugandan cassava brown streak virus* (UCBSV), collectively termed U/CBSVs (Mbanzibwa *et al.*, 2011; Monger *et al.*, 2001a,b; Winter *et al.*, 2010; Amisse *et al.*, 2019). In cassava, U/CBSVs cause foliar chlorosis and root necrosis, and can cause yield losses of up to 100% in susceptible varieties (Kaweesi *et al.*, 2014). Despite the importance of U/CBSVs, relatively little is understood about their fundamental biology and gene functions.

U/CBSVs belong to the *Ipomovirus* genus of the *Potyviridae* family; both viral species share several unusual genome features in that they encode a P1 protein, lack a HC‐Pro protein, and encode Maf/Ham1‐like proteins (Mbanzibwa *et al.*, 2009). Maf/Ham1 proteins belong to the inosine triphosphate (ITP) pyrophosphohydrolase (ITPase) protein family, which in prokaryotes, eukaryotes, and archaea function to specifically hydrolyse non‐canonical, mutagenic nucleotide triphosphates (NTPs) and thereby reduce mutation rates (Simone *et al.*, 2013; Waisertreiger *et al.*, 2010; Zamzami *et al.*, 2013). The non‐canonical NTPs xanthine and inosine triphosphate (XTP/ITP) are formed as by‐products of purine NTP biosynthesis or through oxidative deamination of canonical purine NTPs (Simone *et al.*, 2013). If incorporated into nucleic acid, XTP and ITP can cause deleterious mispairing mutations, nucleic acid strand breaks, and recombinations (Budke and Kuzminov, 2010; Burgis *et al.*, 2003; Simone *et al.*, 2013). ITPase proteins hydrolyse the pyrophosphate bonds in XTP and ITP to release xanthine and inosine monophosphates (XMP/IMP), respectively, and a pyrophosphate (PPi) molecule. This prevents XTP/ITP incorporation into nucleic acid and thereby reduces mutation rates. U/CBSV Ham1 proteins contain the highly conserved serine‐histidine‐arginine (SHR) signature motif, which in the human ITPase is involved with substrate binding and specificity (Gall *et al.*, 2013; Stenmark *et al.*, 2007).

*Potyviridae* genomes are composed of single‐stranded, positive‐sense RNA which are replicated by viral RNA‐dependent RNA polymerase (RdRp). Compared with DNA polymerases, RdRps have relatively low fidelity and lack proof‐reading (Andino and Domingo, 2015; Duffy *et al.*, 2008). This results in populations of closely related viral genome variants, referred to as quasi‐species, that can rapidly adapt to changes in host environments (Andino and Domingo, 2015; Holmes, 2003). It has been reported that RNA viral mutation rates typically exist close to a critical error threshold, above which error catastrophe occurs whereby lethal numbers of mutations accumulate during each round of viral replication, causing a dramatic loss of viral viability (Crotty *et al.*, 2001; Holmes, 2003). RNA viruses must therefore balance maximizing sequence diversity to enhance adaptability whilst conserving key sequences required for multiple essential functions. Proteins with conserved Maf/Ham1 domains are not commonly found in viral genomes and have only been reported in two other viral species that also infect euphorbia hosts: *Euphorbia ringspot virus* (EuRV: *Potyvirus* genus, *Potyviridae* family) and *Cassava torrado‐like virus* (CsTLV: *Torrado* genus, *Secoviridae* family (Knierim *et al.*, 2017; Jiménez Polo, 2018)*.* Previously, it has been hypothesised that Ham1‐like proteins in U/CBSV genomes may function to reduce viral mutation rates below a critical error threshold during infection to retain viral viability over long‐term infections (Mbanzibwa *et al.*, 2009). However, this function for U/CBSV Ham1 proteins is currently uncharacterized.

In addition to gene function, the U/CBSV genome sequence determinants that are associated with necrosis development during infection are unknown. Typically, compared with UCBSV, CBSV causes more severe root necrosis in cassava and accumulates to higher titres (Kaweesi *et al.*, 2014; Ogwok *et al.*, 2014; Winter *et al.*, 2010). In the model host *Nicotiana benthamiana*, CBSV tends to cause extremely severe systemic necrosis that results in plant death, whereas UCBSV tends to cause milder mosaic symptoms (Mohammed *et al.*, 2012; Winter *et al.*, 2010). UCBSV and CBSV Ham1 proteins typically share only 47% amino acid identity, suggesting that this region may be associated with differences in symptom development and viral accumulation during infection (Mbanzibwa *et al.*, 2011; Winter *et al.*, 2010).

In this study, we performed *in vitro* enzyme assays to investigate the ability of CBSV and UCBSV Ham1 proteins to selectively hydrolyse non‐canonical, mutagenic NTPs. We then performed three sets of experiments to test the ability of U/CBSV Ham1 proteins to provide mutagenic protection. First, 5‐fluorouracil resistance assays were performed in yeast overexpressing the U/CBSV Ham1 proteins. Second, transgenic *N. tabacum* lines overexpressing the CBSV Ham1 sequence were infected with *Potato virus Y* (PVY) and *Tobacco mosaic virus* (TMV) and viral genome diversity was measured using deep‐sequencing. Third, the recently constructed CBSV_Tanza infectious clone (IC) (Duff‐Farrier *et al*., 2018) was used to construct the CBSV_HKO IC, containing a Ham1 deletion. Deep‐sequencing was then performed to compare viral genome diversity during CBSV_HKO with wild‐type CBSV infections of *N. benthamiana*. Finally, we investigated the association of the Ham1 region with symptom development and viral accumulation. Our findings shed light on potential U/CBSV Ham1 protein functions that will be useful for further studies into the fundamental biology of these devastating viruses that could lead to novel antiviral strategies.

## Results

### ITPase motifs are highly conserved in CBSV and UCBSV Ham1 sequences

To compare the Ham1 proteins, the CBSV_Tanza and UCBSV_Kikombe Ham1 amino acid sequences were aligned with seven CBSV and seven UCBSV isolates from the NCBI database. This demonstrated that both the CBSV_Tanza and UCBSV_Kikombe Ham1 proteins are 226 amino acids in length, located between the NIb and coat protein (CP) regions. Differences were found in the proteolytic cleavage sequences at the N‐terminus of CBSV and UCBSV Ham1 proteins. Whereas CBSV Ham1 proteins are predicted to be cleaved from the NIb peptide at the cleavage sequence I‐D‐L‐Q‐/V, for UCBSV Ham1 proteins the sequence is V‐D‐T‐Q‐/T, which suggests differential polyprotein processing of the two viral species (Fig. S1). The CBSV_Tanza and UCBSV_Kikombe Ham1 sequences were found to share 46% amino acid identity, which is comparable to the identities of CBSV and UCBSV Ham1 proteins previously reported (Mbanzibwa *et al.*, 2009; Winter *et al.*, 2010). To investigate the level of ITPase conservation in U/CBSV Ham1 proteins, 12 CBSV and 20 UCBSV Ham1 sequences were aligned. Conserved ITPase motifs were identified in the Ham1 sequences in all isolates at amino acid positions: Ser192, His193, and Arg194 (Fig. S2). The ExPASy ProtParam tool was used to predict the molecular weights for the Ham1 proteins according to their amino acid sequences. The CBSV_Tanza and UCBSV_Kikombe Ham1 proteins are predicted to be 25.5 and 25.1 kDa in size, respectively.

### U/CBSV Ham1 proteins have *in vitro* ITPase activities

To determine whether the CBSV_Tanza and UCBSV_Kikombe Ham1 proteins have ITPase activity, protein expression and *in vitro* enzyme assays were performed. Histidine‐tagged Ham1 proteins were expressed in *Escherichia coli* and purified using nickel chromatography columns. Purification of the Ham1 proteins was confirmed by SDS‐PAGE analysis, which demonstrated that homogenous proteins of approximately 25 kDa had been purified, corresponding to the expected size of the CBSV and UCBSV Ham1 proteins (Fig. S3). The concentrations of the purified CBSV and UCBSV Ham1 proteins were estimated to be 0.5 and 0.3 mg/mL, respectively, according to Bradford assay. To detect ITPase activity, enzyme assays were performed whereby 1.3 μg of the CBSV_Tanza or UCBSV_Kikombe Ham1 proteins were incubated with 0.2 mM non‐canonical NTPs: XTP and dITP, and eight canonical ribose and deoxyribose‐NTPs in coupled reactions with 0.1 units of yeast inorganic pyrophosphatase (Thermo Fisher Scientific), according to Lin *et al.*, (2001). Phosphate (Pi) concentration was measured using the PiColourLock kit (Innova Biosciences) recording absorbance values in an iMark Microplate Absorbance Reader microtitre plate reader (Bio‐Rad) with a 655 nm filter. Phosphate concentrations were calculated from A_655_ nm readings using linear regression of known phosphate concentrations.

**Figure 1:**
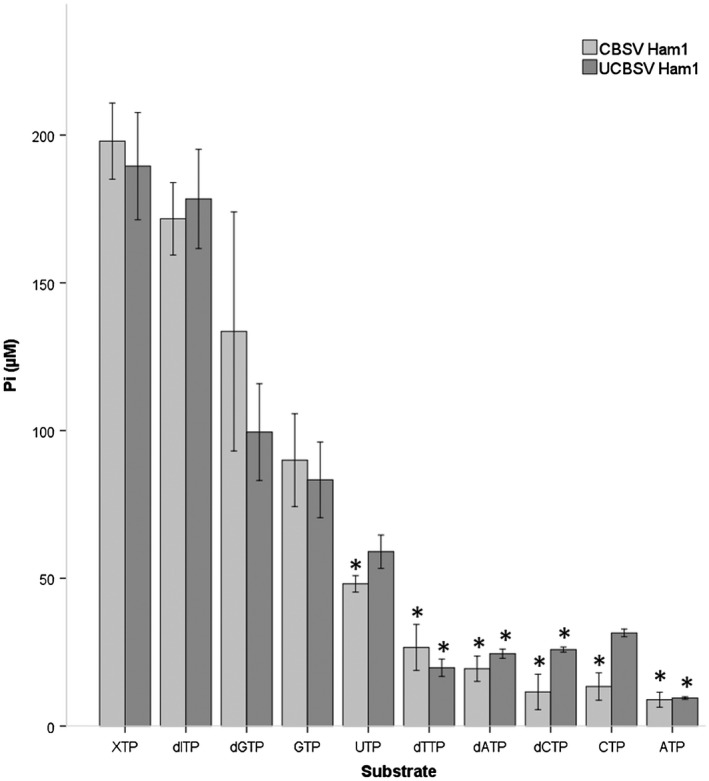
CBSV_Tanza and UCBSV_Kikombe Ham1 proteins have higher ITPase activities with non‐canonical, mutagenic NTPs XTP and ITP, compared with canonical NTPs. Purified CBSV_Tanza and UCBSV_Kikombe Ham1 proteins (1.3 μg) were incubated with 0.2 mM substrates at 37 °C for 20 min in 50 mM Tris‐HCl, pH 8.5, 1 mM DTT, and 50 mM MgCl_2_, according to Lin *et al*. (2001). Activity was measured colourimetrically according to the Experimental Procedures section. One‐way Games–Howell ANOVA analyses demonstrated that the CBSV Ham1 protein has significantly higher activities with the non‐canonical nucleotide triphosphates XTP and dITP compared to canonical nucleotide triphosphates UTP, dTTP, dATP, dCTP, CTP and ATP (*P < 0.05) and that the UCBSV Ham1 protein has significantly higher activities with XTP and dITP compared to dTTP, dATP dCTP, and ATP (*P < 0.05). Results are provided in Tables S1–S2. Each result is the mean phosphate concentration from three separate experiments (*n* = 3) ± SE.

Pi concentrations were negligible in negative control reactions whereby (1) no Ham1 protein was added, (2) bovine serum albumin control protein was added or (3) no yeast inorganic pyrophosphatase was added. Both the CBSV and UCBSV Ham1 proteins showed significantly higher activity with XTP and dITP, followed by dGTP and GTP (Fig. 1). We found that the CBSV Ham1 protein has significantly higher activities with the non‐canonical NTPs XTP and dITP compared to canonical NTPs: UTP, dTTP, dATP, dCTP, CTP, and ATP (*P* < 0.05), and the UCBSV Ham1 protein has significantly higher activities with XTP and dITP compared to dTTP, dATP, dCTP and ATP (*P* < 0.05) (Tables [Link], [Link]). To test whether CBSV Ham1 ITPase activity could be heat inactivated, the protein was heated to 95 °C for 10 min and then added to reactions with dITP. Phosphate concentrations were 97% lower in reactions with heat‐inactivated protein, indicating that heat denaturation abolishes U/CBSV Ham1 ITPase activity (Fig. S4).

### Overexpression of U/CBSV Ham1 proteins in yeast does not provide resistance to 5‐fluorouracil

To test whether CBSV_Tanza and UCBSV_Kikombe Ham1 proteins can protect against mutagenic NTPs *in vivo*, resistance assays were performed in *Saccharomyces cerevisi* *ae* (yeast). Carlsson *et al. *(2013) have previously shown that overexpression of the Ham1 gene in yeast protects against the exogenous supply of the non‐canonical, mutagenic pyrimidine nucleoside 5‐fluorouracil (5‐FU). The yeast Ham1 gene can protect against several non‐canonical nucleosides, including 6‐*N*‐hydroxylaminopurine (Kozmin *et al.*, [Link]; Noskov *et al.*, 1996), 5‐fluorocytosine, and 6‐azauracil (Carlsson *et al.*, 2013). To investigate whether U/CBSV Ham1 proteins can also provide protection from 5‐FU, resistance assays were performed. The Ham1 sequences from CBSV_ Nampula (NCBI: MG019915), CBSV_Tanza (NCBI: MG570022), UCBSV_Kikombe (NCBI: KX753356.1), and yeast (NCBI: 853532) were cloned into pYES2 and transformed into the wild‐type yeast BY4742 strain. Transformant yeast were cultured in liquid Yeast Synthetic Dropout Media (YSDM: 1.7 g/L yeast nitrogen base, 5 g/L ammonium sulphate, and 0.77 g/L yeast uracil dropout mix (Sigma‐Aldrich)), supplemented with 2% galactose to induce Ham1 overexpression and harvested at early log‐phase. Transformant yeast were then plated onto YSDM agar plates, supplemented with 2% galactose and 10 µg/mL 5‐FU, and after 72 h colonies were assessed for growth (Fig. S5). Liquid growth assays were also performed whereby yeast growth was compared in YSDM liquid media supplemented with 2% galactose and 10 µg/mL 5‐FU (Fig. S6). In both plate and liquid assays, yeast transformed with U/CBSV Ham1 sequences showed low levels of growth, comparable to the negative control (empty pYES2), whereas transformants with the yeast Ham1 gene showed relatively high levels of growth. This suggests that unlike the yeast Ham1, the U/CBSV Ham1 proteins are unable to provide protection from mutagenic 5‐FU and so may not function to reduce mutation rates.

### Effects of CBSV Ham1 on viral mutation rates during infections

**Figure 2:**
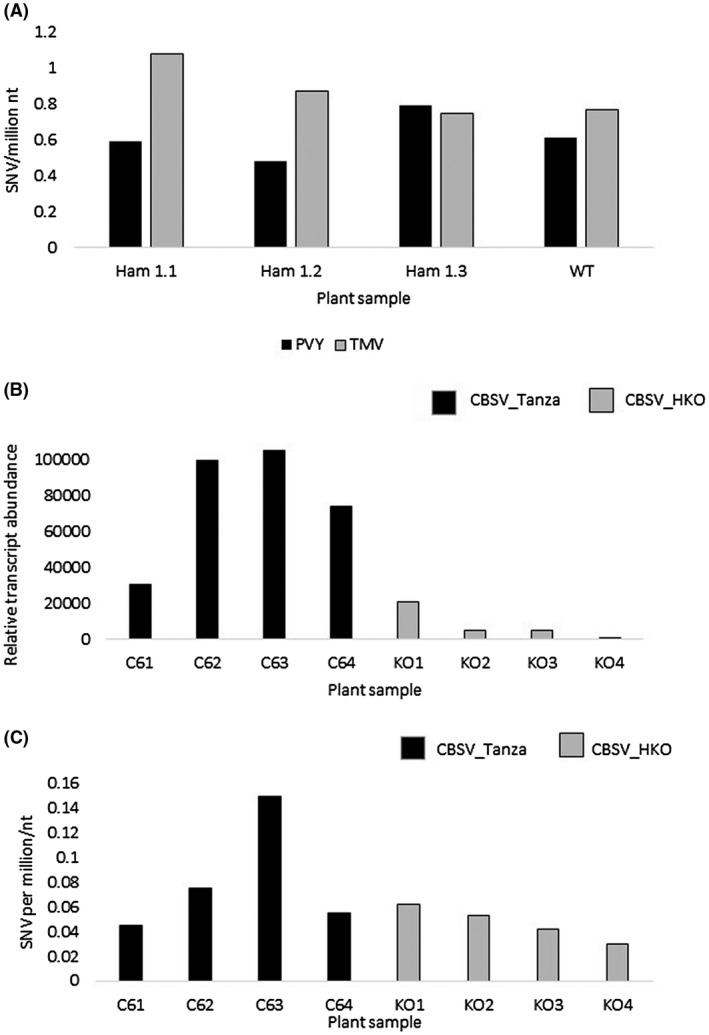
(A) Results from the deep‐sequencing experiment indicate that the transgenic expression of CBSV Ham1 in *Nicotiana tabacum* does not significantly affect PVY or TMV genome diversity. The number of single nucleotide variations (SNVs) per million nucleotides of aligned sequence reads from PVY and TMV infections of wild‐type (WT) *N. tabacum* and three transgenic *N. tabacum* lines expressing the CBSV_Nampula Ham1 sequence (Ham 1.1, Ham 1.2, and Ham 1.3) are shown. (B) qPCR of relative CBSV transcript abundance in CBSV_Tanza infections (C61–4) and CBSV_HKO infections (KO1–4) used in the deep‐sequencing experiment. (C) Results from the deep‐sequencing experiment indicate that the deletion of CBSV Ham1 does not significantly increase CBSV genome diversity. The number of SNVs per million nucleotides of aligned sequenced reads from CBSV_Tanza infections (C61–4) and CBSV_HKO infections (KO1–4) are shown.

Experiments were then performed to test whether the ITPase activities of U/CBSV Ham1 proteins result in lower viral mutation rates during infections. Wild‐type *Nicotiana tabacum* and transgenic *N. tabacum* lines expressing CBSV_Nampula Ham1 were infected with TMV and PVY, and deep‐sequencing was performed on viral amplicons to compare the diversity of viral genomes. To generate transgenic lines, *N. tabacum* was transformed with an expression vector containing the CBSV_Nampula Ham1 sequence (NCBI: MG019915). Expression of the transgene was confirmed in three separate lines by quantitative real‐time PCR (qPCR) (Table S5). Wild‐type and transgenic *N. tabacum* plants were inoculated with TMV (genus: *Tobamovirus*) and PVY (genus: *Potyvirus*). At 14 days post‐inoculation (dpi), RT‐PCR was performed targeting two 500 bp regions of each viral genome. For TMV, the 126 kDa replicase and 54 kDa replicase regions were targeted and for PVY, the NIb and CP were targeted. Next‐generation sequencing (NGS) was performed on these amplicons and sequence reads were aligned to the reference sequences with a read depth of 100 000 reads. The LoFreq algorithm was used to detect viral variants which occur at a low frequency (Wilm *et al.*, 2012). No differences were found in the number of single nucleotide variants (SNVs) in TMV and PVY RT‐PCR amplicons from Ham1‐transgenic and wild‐type *N. tabacum* plants (Fig. 2A). This indicates that CBSV Ham1 protein does not reduce TMV or PVY mutation rates in *N. tabacum*.

To investigate in greater depth whether the CBSV Ham1 protein specifically reduces the CBSV mutation rate, the complete Ham1 sequence was deleted from the CBSV_Tanza IC (Duff‐Farrier *et al.*, 2019) to produce the CBSV_Tanza Ham1 knockout IC (CBSV_HKO). *N. benthamiana* plants were then infected with either CBSV_Tanza or CBSV_HKO. At 10 dpi, RT‐PCR was performed targeting 1200 bp of CBSV coat protein sequence. As before NGS and LoFreq analysis was performed on aligned reads. To determine whether the samples contained different viral titres, qPCR was performed which demonstrated that viral transcript abundance was higher in the wild‐type CBSV_Tanza infections than in CBSV_HKO infections (Fig. 2B). Therefore, to account for SNVs in viral amplicons that are due to higher viral titres rather than changes to mutation rates, viral titres were included in analyses. A one‐way ANOVA test found no significant difference in the number of SNVs in sequencing reads from wild‐type CBSV_Tanza and CBSV_HKO, whilst accounting for variance in viral titres (Fig. 2C), F(1, 0.008) = 1.423, *P* = 0.286. This indicates that the CBSV Ham1 does not reduce the CBSV mutation rate in *N. benthamiana*.

### Association of U/CBSV Ham1 proteins with necrosis development and viral accumulation

**Figure 3:**
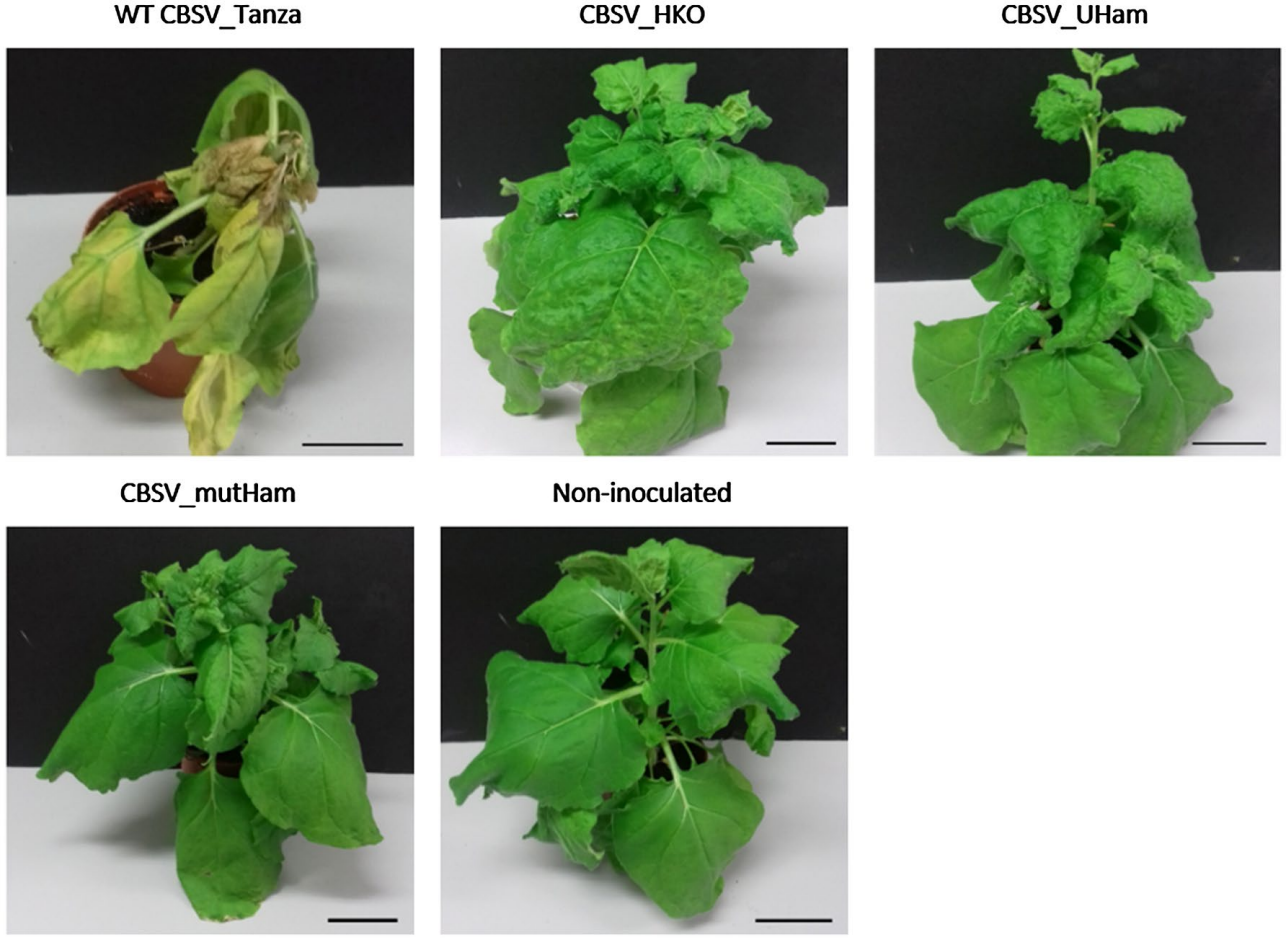
CBSV Ham1 is associated with the development of necrosis during *Nicotiana benthamiana* infection. Symptom development during *N. benthamiana* infections with CBSV_Tanza infectious clones at 18 days post‐inoculation (dpi). Wild‐type (WT) CBSV_Tanza infections develop severe systemic necrosis, chlorosis, stunting, and plant death. Infections with the Ham1 knockout infectious clone: CBSV_HKO, UCBSV Ham1 replacement: CBSV_UHam and mutated SHR motif: CBSV_mutHam develop systemic leaf curling, chlorotic mottling and stunting at 10–18 dpi, compared with non‐inoculated plants. This suggests that the CBSV Ham1 protein is not essential for infection and is associated with necrosis development during *N. benthamiana* infection. Scale bar = 5 cm.

*N. benthamiana* infected with CBSV_HKO developed dramatically different symptoms compared with the IC of wild‐type CBSV_Tanza (Fig. 3). Whilst wild‐type CBSV_Tanza infections of *N. benthamiana* developed severe necrosis and plant death at 10–14 dpi, CBSV_HKO infections did not develop necrosis but instead developed strong systemic leaf curling, chlorotic mottling, and stunting at 10–18 dpi (Fig. 3). This suggests that the CBSV Ham1 is not essential for infection and is associated with necrosis development during infection of *N. benthamiana*.

**Figure 4:**
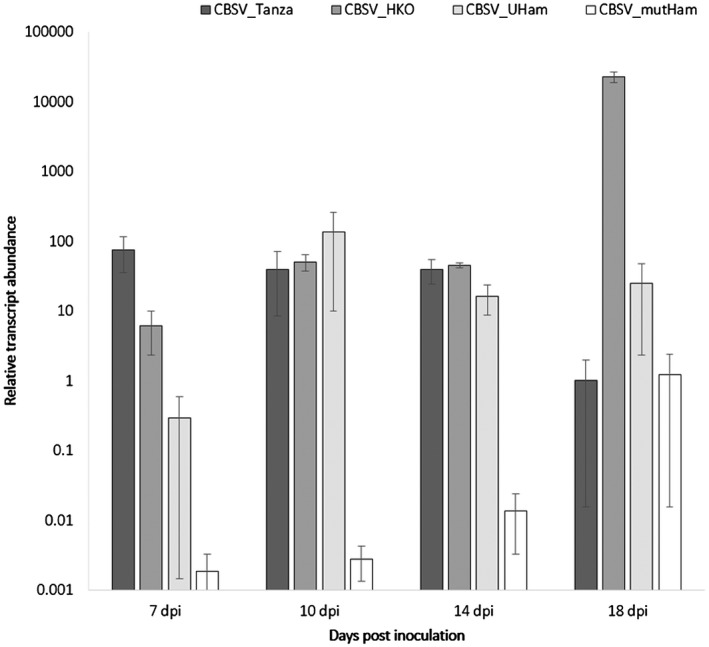
CBSV Ham1 is involved with viral accumulation during *Nicotiana benthamiana* infections. *N. benthamiana* was agroinfiltrated with the following infectious clones: wild‐type CBSV_Tanza, Ham1 deletion (CBSV_HKO), mutated Ham1 ITPase SHR motif (CBSV_mutHam), and the chimera infectious clone containing a UCBSV Ham1 swap (CBSV_UHam). qPCR was performed at 7, 10, 14 and 18 days post‐inoculation (dpi). Each result is the mean from three replicate plants (*n* = 3) ± SE. Results demonstrate that CBSV_Tanza accumulates to higher titres during early infection (7 dpi) compared with CBSV_HKO, CBSV_mutHam and CBSV_UHam, suggesting that Ham1 may be involved with early viral accumulation. Results were consistent in three separate experiments.

To further investigate the association of the CBSV_Tanza Ham1 protein with necrosis development, additional modifications to the CBSV_Tanza IC were performed. First, to test whether the development of necrosis during CBSV_Tanza infections of *N. benthamiana* is specifically associated with the ITPase motifs within the CBSV Ham1 sequence, the CBSV_mutHam IC was constructed whereby the positively charged SH(+)R(+)motif was mutated to neutrally charged serine‐alanine‐alanine (SAA). This mutation is predicted to alter electrostatic interactions of the CBSV Ham1 protein and thereby affect function. Gall *et al*., (2013) previously demonstrated that mutating the SHR motif to SAA in the human ITPase protein abolishes ITP substrate specificity or activity. Second, to also test whether the CBSV_Tanza and UCBSV_Kikombe Ham1 proteins are associated with differential symptom development during *N. benthamiana* infections, the chimeric CBSV_UHam IC was constructed, consisting of the CBSV_Tanza genome with a UCBSV_Kikombe Ham1 sequence replacement (Fig. S7). *N. benthamiana* plants were then agroinfiltrated with the modified ICs (CBSV_HKO, CBSV_mutHam and CBSV_UHam) so that symptoms and viral titres could be compared with wild‐type IC CBSV_Tanza infections. *N. benthamiana* infected with the different ICs developed a range of symptoms, as illustrated in Fig. 3. As already seen with CBSV_HKO, infections with CBSV_mutHam and CBSV_UHam also lack necrosis and develop leaf curling, chlorotic mottling, and stunting at 12–18 dpi. This further suggests that the CBSV Ham1 protein is associated with necrosis development during infection of *N. benthamiana*. To identify the effect of the manipulations on viral accumulation, qPCR was performed throughout infection. This revealed that CBSV_Tanza accumulates to higher titres during early infection (7 dpi) compared with CBSV_HKO, CBSV_mutHam and CBSV_UHam, suggesting that Ham1 may be involved with early viral accumulation (Fig. 4). Later in the infection (10–18 dpi), CBSV_UHam accumulates to levels similar to CBSV_Tanza, suggesting potential complementation of the UCBSV Ham1 protein with CBSV. Titres remained low throughout CBSV_mutHam infections, demonstrating that mutating the SHR motif in the viral Ham1 protein has a large effect on viral accumulation. CBSV_HKO titres accumulated to high levels at 18 dpi, indicating that the Ham1 protein is not required for high levels of viral replication later in infection of *N. benthamiana*.

## Discussion

This study has demonstrated that CBSV and UCBSV Ham1 proteins have ITPase activities with non‐canonical, mutagenic NTPs. While this activity was detected *in vitro*, the *in vivo* functions of these proteins remain unclear. The ability of the CBSV IC lacking a Ham1 (CBSV_HKO) to infect and accumulate to high levels in *N. benthamiana* is surprising as RNA viruses typically have highly streamlined genomes that encode a small number of proteins with multiple, indispensable functions (Holmes, 2003). Genome regions that do not serve advantageous functions tend to be rapidly lost from viral genomes during infections. This is demonstrated by the rapid deletion of marker‐gene sequences during infections with tagged viral ICs (Arazi *et al.*, 2001; Beauchemin *et al.*, 2005; Dawson *et al.*, 1989; Guo *et al.*, 1998). Willemsen *et al., *(2017) have also demonstrated that exogenous sequences are only maintained in *Tobacco etch virus* (TEV) genomes during infection if they provide advantageous functions for TEV. As all reported U/CBSV genome sequences encode Ham1 proteins with highly conserved ITPase motifs, they must serve advantageous functions during infection and/or transmission. We suggest that although the CBSV Ham1 protein was dispensable for CBSV infection of *N. benthamiana* in this study, further studies are required to determine whether U/CBSV Ham1 proteins are required specifically during cassava infections and/or vector transmission.

This is the first report that CBSV and UCBSV Ham1 proteins have ITPase activities and that these are significantly higher with non‐canonical compared with canonical NTPs. The U/CBSV Ham1 ITPase activities are similar to those of previously characterized ITPase proteins from the archaea thermophile *Methanococcus jannaschii* (Cho *et al.*, 1999), human (Lin *et al.*, 2001), and *E. coli* (Burgis *et al.*, 2003; Zheng *et al.*, 2005). Compared with these proteins, U/CBSV Ham1 proteins have higher activities with the canonical nucleotide (d)GTP. The reasons for this may include lower selective pressure on U/CBSV Ham1s to maintain binding cleft structures that are unfavourable to (d)GTP binding or disruption of host processes, such as G‐protein defence signalling (Brenya *et al.*, 2016; Trusov and Botella, 2016), host DNA replication, and expression or membrane trafficking (Inada and Ueda, 2014), which requires further investigation.

Despite finding that U/CBSV Ham1 proteins have ITPase activities with non‐canonical NTPs *in vitro*, we found no evidence of mutagenic protection in 5‐FU resistance assays in yeast. This result is difficult to explain but perhaps indicates that the viral ITPase does not target the same subcellular location as the native yeast Ham1. We were also unable to detect any significant decreases in viral genome variation during PVY and TMV infections of CBSV Ham1 transgenic *N. tabacum* lines or increases in CBSV genome variation during CBSV_HKO infections of *N. benthamiana*. These preliminary results indicate that U/CBSV Ham1 proteins do not lower viral mutation rates during infections of *Nicotiana* hosts. However, the functions of U/CBSV Ham1 proteins may be complex and depend on other factors, including host and environment. Studies have found that different host environments can affect viral mutation rates, for instance *Cucumber mosaic virus* replicates with a lower fidelity during infections of pepper compared with tobacco (Pita *et al.*, 2007). Therefore U/CBSV Ham1 proteins may have a bigger effect on viral mutation rates specifically during replication in cassava. U/CBSV Ham1 proteins may also play important functions during replication in wild hosts that can be found in close proximity to CBSD‐affected cassava fields (Amisse *et al.*, 2018). If viral Ham1 sequences are found to serve as a euphorbia or wild host adaptation, they could be targeted in antiviral strategies such as post‐transcriptional gene silencing.

In this study, CBSV IC manipulations demonstrated that the CBSV Ham1 is associated with the development of necrosis in *N. benthamiana*. Infections with CBSV ICs containing a deletion of the Ham1 sequence (CBSV_HKO), mutated SHR ITPase motif (CBSV_mutHam), and a UCBSV Ham1 chimera (CBSV_UHam) did not develop necrosis in *N. benthamiana*, but instead developed systemic leaf curling, chlorotic mottling, and stunting. The CBSV Ham1 ITPase activity may be directly associated with necrosis development, but as *in vitro* enzyme assays demonstrated that the CBSV and UCBSV Ham1 proteins have similar ITPase activities and the CBSV_UHam infections lack necrosis, there may be alternative explanations. For instance, the folded CBSV Ham1 protein may be recognized by the host, which triggers severe, systemic necrosis. Examples of this in other systems include recognition of the *Soybean mosaic virus* P3 protein by the host Rsv1 protein, which leads to the induction of a lethal systemic hypersensitive response (Hajimorad *et al.*, 2005). It should be noted that although the CBSV Ham1 protein is associated with the development of necrosis in *N. benthamiana*, this may be different to root necrosis development in cassava. We suggest that the ICs developed in this study should be used to investigate whether the CBSV Ham1 region is associated with root necrosis during cassava infections.

In addition to necrosis in *N. benthamiana*, the CBSV Ham1 protein also appears to be involved with viral accumulation during early infection. Analysis of CBSV transcript abundance by qPCR indicated that during early infection CBSV_Tanza accumulated to higher levels than CBSV_KO. However, later in infection, CBSV_HKO increased to much higher levels than CBSV_Tanza, which is likely to be due to the lack of necrosis that limits wild‐type CBSV_Tanza accumulation. Interestingly, CBSV_UHam transcripts accumulated to levels comparable to wild‐type CBSV_Tanza, which may indicate that the UCBSV Ham1 is able to at least partially compensate for the absence of the CBSV Ham1. The potential synergistic and/or antagonist interactions between CBSV and UCBSV proteins are currently poorly understood, despite the frequent occurrence of mixed infections in the field (Kathurima *et al.*, 2016; Mbanzibwa *et al.*, 2011; Ogwok *et al.*, 2014).

In line with previous research, we found that, outside of the conserved ITPase motifs, CBSV and UCBSV Ham1 proteins share relatively low amino acid sequence identity. They have different N‐terminus proteolytic cleavage sequences and so may form different intermediate fusion proteins with different specific functions (Adams *et al.*, 2005), which requires further investigation.

To conclude, we have demonstrated that UCBSV and CBSV Ham1 proteins selectively hydrolyse non‐canonical, mutagenic NTPs *in vitro*. We have also shown that the CBSV_Tanza Ham1 protein is associated with the development of necrosis during infections of *N. benthamiana*. We found no evidence that U/CBSV Ham1 proteins function to reduce viral mutation rates during infections of experimental *Nicotiana* hosts. Further studies are needed to determine the functions of U/CBSV Ham1 proteins during infections of cassava and whether they serve as euphorbia host adaptations. Information on the specific functions of U/CBSV Ham1 proteins during cassava infections could lead to novel antiviral strategies.

## Experimental Procedures

### U/CBSV Ham1 sequence alignments

U/CBSV Ham1 amino acid sequences were downloaded from the NCBI database. Sequences were aligned using the T‐coffee multiple sequence alignment package (http://tcoffee.crg.cat/apps/tcoffee/index.html), according to Notredame *et al., *(2000). Alignments were formatted using the Expasy Boxshade tool (https://embnet.vital-it.ch/software/BOX_form.html).

### U/CBSV Ham1 protein expression and purification

The CBSV_Tanza and UCBSV_Kikombe Ham1 sequences were amplified from the CBSV_Tanza (NCBI: MG570022) and UCBSV_Kikombe (NCBI: KX753356) ICs respectively by PCR using the high‐fidelity Phusion polymerase (Thermo Fisher Scientific). Primers were designed to include 21 bp of overlapping sequence with the POPINF plasmid vector (OPPF‐UK). Primers used to amplify the CBSV_Tanza and UCBSV_Kikombe Ham1 sequences are provided in Table S3. The PCR products encoding the Ham1 sequences were cloned into the POPINF expression vector (OPPF‐UK) using the InFusion cloning kit (Takara Bio USA, Inc.). The POPINF vector contains the 6× histidine tag, which is fused to the 3ʹ of insert sequences. Plasmids were transformed into electrocompetent TOP10 *E. coli* and blue/white screening was used to select colonies containing plasmids with inserts. Transformant colonies were cultured and plasmids were extracted using the GeneJet Plasmid Miniprep Kit (Thermo Fisher Scientific). Plasmids were Sanger sequenced to confirm the presence of Ham1 sequences without mutation in both POPINF plasmids.

For expression, POPINF plasmids were transformed into the *E. coli* strain: BL21‐DE3 (New England Biolabs). *E. coli* cultures were incubated in Luria Broth media containing 100 μg/mL carbenicillin and incubated at 37 °C and 200 rpm until the OD_600_ was approximately 0.4. Protein expression was induced through the addition of 1 mM isopropyl β‐d‐1‐thiogalactopyranoside (IPTG) and cultures were incubated for a further 18 h. The cultures were then centrifuged at 6000 *g* for 30 min at 4 °C; cell pellets were resuspended in the resuspension buffer pH 7.5 (150 mM NaCl, 20 mM Tris‐HCl pH 7.5) and centrifuged as before. The cell pellets were then resuspended in loading buffer pH 7.5 (0.5 M NaCl, 20 mM Tris‐base, 20 mM imidazole, 10% glycerol). Samples were homogenized and lysed using a French pressure cell press (Constant Systems). The lysate was centrifuged at 18 000 *g* for 40 min at 4 °C and the supernatant was filtered using a 0.22 μm syringe filter (Millipore). The lysate was loaded onto a HisTrap FF Crude histidine‐tagged protein purification column (GE Healthcare). A fast‐protein‐liquid chromatography (ÄKTA) machine was used to set up an imidazole concentration gradient of 50% loading to elution buffer pH 7.5 (0.5 M NaCl, 20 mM Tris‐base, 1 M imidazole, 10% glycerol). Fractions corresponding to a peak in UV absorbance were analysed by SDS‐PAGE to confirm the presence of a protein band with the expected size. Fractions containing protein were pooled dialysed using a SnakeSkin 10 kDa dialysis tube (Thermo Fisher Scientific) into the storage buffer pH 7.5 (0.5 M NaCl, 50 mM Tris‐HCl pH 7.5, 1 mM dithiothreitol (DTT), 50 mM MgCl_2_, 20% glycerol). To increase the concentration of the protein, the sample was transferred to a 15 mL Vivaspin column (GE Healthcare) with a molecular weight cut‐off of 10 kDa. *In vitro* ITPase enzyme assays were performed as described in the Results section. Nucleotide triphosphates were obtained from Thermo Fisher Scientific (dGTP, dCTP, dTTP, dATP, dITP), Invitrogen (GTP, CTP, ATP, UTP) and Sigma Aldrich (ITP and XTP).

### 5‐fluorouracil resistance assays in yeast

The *S. cerevisiae* (yeast) Ham1 gene (NCBI: 853532) was amplified by PCR from the wild‐type yeast strain BY4742/ Y10000 (Euroscarf). The U/CBSV Ham1 sequences were amplified by PCR from the CBSV_Tanza, CBSV_Nampula, and UCBSV_Kikombe ICs, respectively, using the high‐fidelity Phusion polymerase (Thermo Fisher Scientific). The Ham1 sequences were cloned into pYES2 plasmids, downstream of the inducible *GAL1* promoter. Plasmids were Sanger sequenced to confirm the presence of *Ham1* sequences without mutation. The plasmids were transformed into the wild‐type yeast strain BY4742 (Y10000). Colony PCR confirmed the presence of the *Ham1* genes in transformant yeast. Plate and liquid growth assays were performed as described in the Results section.

### Reverse transcription PCR

RNA was extracted from *Nicotiana* plants using the E.Z.N.A. Plant RNA kit (Omega Bio‐Tek). Samples were DNase I treated (Thermo Fisher Scientific) and reverse transcription was performed on 1 μg of RNA using the First Strand cDNA synthesis kit (Thermo Fisher Scientific) and the oligo d(T)18 primer. First‐strand cDNA was used as the template in PCRs using high‐fidelity Phusion polymerase (Thermo Fisher Scientific) and primers to target specific regions of the viral genomes.

### Quantitative real‐time PCR

qPCR was performed to detect the expression level of the CBSV_Nampula *Ham1* sequence in transgenic *N. tabacum*. Primers to detect CBSV_Nampula *Ham1* sequence were qPCR_CHam1_Fw: CGAGTAGCTGCTGAACTTGTTGGAG/qPCR_CHam1_Rv: GATATGGCTCCACCAACTTGTATAG. The *Protein phosphatase 2A* (PP2A) (NCBI: TC21939/At1g13320) was used as an endogenous reference gene and targeted using primers designed and validated in Liu *et al. *(2012). qPCR was also performed to calculate relative viral transcript abundance in infected *N. benthamiana* material. The primers used to detect the CBSV_Tanza *coat protein* were qPCR_CBSV_CP_Fw: ACTTCCTAGCCGAAGCACAA/ qPCR_CBSV_CP_Rv: GCACTAACATCCCGCGTAGT. The *N. benthamiana* F‐box gene (NCBI: 24993647/At5g15710) was used as an endogenous reference gene and targeted using primers designed and validated in Liu *et al. *(2012). All primers were validated for amplification efficiency using a 1:10 serial dilution of template; only primers with *R* ^2^ > 0.99 were used. Inspection of the qPCR amplification peaks confirmed single amplification peaks. Reactions were set up using Maxima SYBR Green/ROX (Thermo Fisher Scientific) following the manufacturer’s instructions and using a Stratagene MX3005 thermocycler. The following programme cycle was used: initial denaturation at 95 °C for 10 min, followed by 40 cycles of (denaturation at 95 °C for 15 s, primer annealing at 60 °C for 30 s, and extension at 72 °C for 30 s). Data were acquired during the extension phase. The MxPro software calculated the threshold Ct values. Relative target gene expression in the three replicate plants were then calibrated with three control samples, using the 2^‐ΔΔCt^ method (Livak and Schmittgen, 2001).

### Deep‐sequencing of viral amplicons

Viral amplicons were purified using the GeneJET gel extraction kit (Thermo Fisher Scientific) and used to prepare libraries according to the TruSeq Nano DNA Library Prep protocol (Illumina). Amplicons were fragmented and cleaned using the AmPure XP 0.8 clean‐up procedure and two indexed adapters were ligated to the paired ends of the fragments. PCR amplification was then performed to enrich for fragments containing both paired‐end adaptors. Library sizes and purity were then validated using a DNA 1000 ScreenTape. Subsequently the samples were diluted to 10 pM with 25% PhiX spike‐in and run in a MiSeq instrument using the MiSeq Reagent Kit v3 (2× 75 bp). FASTQ files generated from the MiSeq run were uploaded onto the Partek Flow server. The Illumina adapter sequences and low‐quality reads with phred scores of less than 30 were then trimmed. Reads were aligned in Partek Flow to reference sequences using Bowtie 2‐2.2.5, very‐sensitive‐local pre‐sets. The LoFreq algorithm was used to detect low‐frequency variants (<0.5%) from sequencing errors (Wilm *et al.*, 2012).

### Infectious clone manipulations

Infected plant material and viral ICs were used under the DEFRA license no. 51045/197610/2 and handled according to Brewer *et al. *(2018). IC manipulations were performed through homologous yeast recombination according to the protocol outlined in Duff‐Farrier *et al. *(2015, 2019). Briefly, the CBSV_Tanza IC (NCBI: MG570022) was linearized with the restriction enzyme *Bam* H1 (Thermo Fisher Scientific). Insert fragments were designed to contain 30 bp of homologous sequences at the 5ʹ and 3ʹ ends to enable recombination of overlapping sequences into linearized CBSV_Tanza IC. Insert fragments were amplified by PCR using high‐fidelity Phusion polymerase (Thermo Fisher Scientific). The conserved SHR motif was mutated through site‐directed mutagenesis with primers encoding the SAA mutation. The UCBSV Ham1 sequence was amplified from the UCBSV_Kikombe IC (NCBI: KX753356) and the CBSV NIb–Ham1 proteolytic cleavage sequence was maintained according to the schematic in Fig. S7. All primers used in the construction of ICs are listed in Table S4. The *S. cerevisiae* YPH499 strain was transformed with linearized CBSV_Tanza IC and PCR insert fragments at a ratio of 4:1 according to the Gietz *et al. *(2002) protocol. Transformant cells were plated onto YSDM agar plates and incubated at 28 °C for 48 h. Plasmids were extracted from transformant yeast using the Zymoprep Yeast Plasmid Miniprep II kit (Zymo Research, USA) and electroporated into the *E. coli* strain TOP10 (Thermo Fisher Scientific). Transformant *E. coli* colonies were cultured in LB broth containing 50 µg/mL kanamycin and plasmids were extracted using GeneJet Plasmid Miniprep Kit (Thermo Fisher Scientific). Recombinant plasmids were analysed by restriction digest, PCR, and Sanger sequencing to confirm correct construction.

### Plant cultivation

Wild‐type *N. tabacum* and *N. benthamiana* plants were cultivated from seed produced in‐house and planted in Sinclair all‐purpose potting compost. All plants were grown in controlled growth cabinets at 28 °C with a 16 h/8 h light/dark cycle.

### Generation of transgenic plants

CBSV_Ham1 transgenic *N. tabacum* lines were produced according the Gallois and Marinho (1995) method. Briefly the CBSV_Nampula Ham1 sequence was cloned into a pCambia 2300 expression vector, containing a *Cauliflower mosaic virus* 35S promoter and tNOS terminator. Electrocompetent *Agrobacterium tumefaciens* strain LBA4404 were transformed with the expression vector and grown in 20 mL LB media, supplemented with 50 μg/mL kanamycin and 20 μg/mL rifampicin. Liquid culture was diluted with Murashige and Skoog media (MSO) until OD_600_ = 1. Fully expanded leaves of *N. tabacum* were collected 5 weeks after germination and sterilized by washing in a solution of bleach at 10% v/v for 10 min and rinsed with sterile distilled water for 15 min. Rinsing was performed five times to eliminate residues of bleach from the surface of the leaves. Once leaves were sterilized, they were cut into small discs of 1 cm in diameter and immersed into agrobacterium MSO culture for 20 min. After inoculation, discs were placed on MSO shooting agar plates. Later plates were placed at 25 °C in a photoperiod of 16 h light and 8 h dark for 2 days in order to adapt to the media and start to develop shoots. After the period of adaptation, disc leaves were removed from the first plate and placed on selective MSO shooting media supplemented with 50 μg/mL kanamycin to select transformed plants and 200 μg/mL timentin to eliminate agrobacteria. Leaf discs and callus cultures were transferred onto fresh media every 3 weeks. Plantlet shoots were excised and transferred to MSO rooting agar medium supplemented 50 μg/mL kanamycin and 200 μg/mL timentin. Once roots were established and leaves were fully developed, transgenic lines were assessed for the presence of the transgenic transcript by qPCR. Verified transgenic *N. tabacum* plants were micropropagated by cutting stems with one to two nodes and placed into MSO rooting media.

### Viral infections

*N. tabacum* and *N. benthamiana* plants were infected with CBSV_Tanza ICs through agroinfiltration, according to Voinnet *et al. *(2003). Electrocompetent agrobacterium strain LBA4044 was transformed with the CBSV_Tanza IC plasmids. Transformant colonies were cultured in LB broth containing kanamycin 50 µg/mL and rifampicin 20 µg/mL. After 48 h the starter culture was used to inoculate LB broth containing kanamycin 50 µg/mL, rifampicin 20 µg/mL, 10 mM morpholino ethane sulfonic acid buffer (MES) and 150 µM of acetosyringone, which was cultured for 20 h. The cells were pelleted, washed, and resuspended in infiltration buffer (10 mM MgCl_2_; 10 mM MES and 150 µM acetosyringone) to an OD_600_ of 1.0. The cells were left at room temperature for 5 h. Plants were agroinfiltrated at 4 weeks; the abaxial surface of the youngest fully expanded leaf was infiltrated with 1 mL of agrobacterium suspension. *N. tabacum* plants were mechanically inoculated with PVY and TMV infected leaf material that was ground in liquid nitrogen and suspended in sterile deionized water. The adaxial surface of the youngest fully expanded leaf was dusted with 600‐mesh carborundum powder (Thermo Fisher Scientific) and grounded tissue gently applied.

## Supporting information


**Fig. S1** Relevant sections of a T‐coffee alignment of eight CBSV and eight UCBSV Ham1 amino acid sequences showing differences in proteolytic cleavage sequences. The junctions where the NIb and CP proteins end are highlighted in yellow. N‐terminus Ham1 proteolytic cleavage sequences between the NIb–Ham1 proteins are different in CBSV isolates (green) and UCBSV isolates (pink), indicating potential differences in proteolytic processing. The C‐terminus Ham1 proteolytic cleavage sequence between the Ham1–CP proteins is conserved in CBSV and UCBSV sequences (blue). The cleavage sites where the are shown in yellow; highly conserved regions (>90%) are highlighted in black. Sequences were obtained from the NCBI database; accession numbers are provided for each sequence.

**Fig. S2** Relevant section of T‐coffee alignment of 12 CBSV and 20 UCBSV Ham1 amino acid sequences. The ITPase signature serine‐histidine‐arginine (SHR) motif is highly conserved and was found in all sequences at positions 192–194 (yellow). Highly conserved regions (>90%) are highlighted in black. Sequences were obtained from the NCBI database; accession numbers are provided for each sequence.

**Fig. S3** SDS‐PAGE of purified CBSV_Tanza (gel A) and UCBSV_Kikombe (gel B) Ham1 proteins (25 kDa). Lanes in gel A correspond to separate fractions B4–B13 that were eluted from the AKTA machine during protein purification at a range of imidazole concentrations. Lane D in gel B refers to the UCBSV Ham1 protein which had been dialysed into the storage buffer. To prepare the protein samples for loading, 10 μL of loading buffer (4% SDS, 0.25 M Tris.HCl pH 6.8, 20% glycerol, 0.004% bromophenol blue, 10% β‐mercaptoethanol), 1 μL of protein sample, and 9 μL of water were mixed and heated at 95 °C for 5 min. A TruPAGE Precast Gel (Sigma Aldrich) was loaded with 10 μL of each prepared sample and 10 μL of PageRuler Protein Ladder (Thermo Fisher Scientific). The gel was run at 220 V for 40 min. The gel was stained with 20 mL InstantBlue Protein Stain (Sigma Aldrich) and analysed under white light using the ChemDoc Bio Rad System. Images were taken using the Quantity One 1D software (Bio‐Rad).

**Fig. S4** Enzyme assay results from the heat inactivation experiment to test for loss of CBSV_Tanza Ham1 ITPase activity. Incubation of 0.2 mM dITP with active CBSV_Tanza Ham1 protein (1.3 μg) resulted in a phosphate concentration of 136 μM. Heating the CBSV_Tanza Ham1 at 95 °C for 10 min to 1 h resulted in a 41–43% reduction in phosphate concentration, indicating inactivation of its pyrophosphohydrolase activity. Control assays were set where BSA protein (1.3 μg) was added, which produced a comparable phosphate concentration to assays where CBSV_Tanza Ham1 had been heat inactivated, indicating that BSA could be used as a control for addition of protein to assay samples. Low background phosphate concentrations were found in negative controls: (1) containing 0.2 mM dITP in reaction buffer with the addition of 0.1 units of yeast inorganic pyrophosphatase, (2) 0.2 mM dITP in reaction buffer, and (3) water.

**Fig. S5** 5‐FU resistance agar plate growth assays. The wild‐type yeast strain BY4742 was transformed with pYES2 plasmids containing *Ham1* sequences from CBSV_Nampula, CBSV_Tanza, UCBSV_Kikombe and yeast. Transformant yeast was cultured and plated onto SD media as ten‐fold serial dilutions onto test plates containing 2% galactose and 10 µg/mL 5‐FU or control plates containing 2% galactose only. Colony growth was imaged after 72 h. Results were consistent in three separate experiments.

**Fig. S6** 5‐FU resistance liquid growth assays. The wild‐type yeast strain BY4742 was transformed with pYES2 plasmids containing Ham1 sequences from CBSV_Nampula, CBSV_Tanza, UCBSV_Kikombe and yeast. Transformant yeast was cultured in liquid SD media containing 2  galactose and 10 µg/mL 5‐FU. The cell density (OD_600_) was taken at 4, 8, 12, 24, 48 and 72 h. Yeast transformed with yeast Ham1 sequence demonstrated relatively high levels of growth. Yeast transformed with U/CBSV Ham1 sequences showed low levels of growth, comparable to the negative control (empty pYES2 plasmid). This indicates that unlike the yeast Ham1 sequence, the U/CBSV Ham1 sequences were unable to protect against mutagenic 5‐FU. Each result is the mean OD_600_ value from three replicate samples (*n* = 3) ± SE. Results were consistent in three separate experiments.

**Fig. S7** The replacement of CBSV_Tanza Ham1 sequence (blue) with UCBSV Ham1 sequence (red) in the CBSV_UHam1 IC. To ensure proteolytic cleavage of UCBSV Ham1 sequence from the CBSV Tanza polyprotein the NIb‐Ham1 protease cleavage sequence IDLQV was maintained at the start of the UCBSV Ham1 sequence TKD.

**Table S1** Games–Howell one‐way ANOVA tests to compare mean phosphate concentration in enzyme assay reactions with CBSV_Tanza Ham1 incubated with the non‐canonical nucleotides XTP and dITP, and a range of canonical nucleotides.

**Table S2** Games–Howell one‐way ANOVA tests to compare mean phosphate concentration in enzyme assay reactions with UCBSV_Kikombe Ham1 incubated with the non‐canonical nucleotides XTP and dITP, and a range of canonical nucleotides.

**Table S3** Primers used to amplify CBSV and UCBSV Ham1 sequences during cloning into the POPINF vector. Sequences overlapping with the POPINF vector are shown in red.

**Table S4** Primers used to amplify PCR fragments to construct the CBSV_mutHam and CBSV_UHam ICs. The nucleotide sequence encoding the SHR to SAA mutation is shown in blue. The nucleotide sequence encoding the UCBSV Ham1 is shown in red.

**Table S5** Relative expression of the CBSV_Nampula Ham1 gene in three transgenic *Nicotiana tabacum* lines compared with wild‐type; detected by qPCR as described in the Experimental Procedures section.
