# Supplementary material for: Cassava brown streak virus Ham1 protein hydrolyses mutagenic nucleotides and is a necrosis determinant
Source: Mol Plant Pathol. 2019 Jun 1;20(8):1080–92. doi: 10.1111/mpp.12813 (PMC6640186; doi:10.1111/mpp.12813)
Supplement: Supplementary file 1 — Fig. S1 Relevant sections of a T‐coffee alignment of eight CBSV and eight UCBSV Ham1 amino acid sequences showing differences in proteolytic cleavage sequences. The junctions where the NIb and CP proteins end are highlighted in yellow. N‐terminus Ham1 proteolytic cleavage sequences between the NIb–Ham1 proteins are different in CBSV isolates (green) and UCBSV isolates (pink), indicating potential differences in proteolytic processing. The C‐terminus Ham1 proteolytic cleavage sequence between the Ham1–CP proteins is conserved in CBSV and UCBSV sequences (blue). The cleavage sites where the are shown in yellow; highly conserved regions (>90%) are highlighted in black. Sequences were obtained from the NCBI database; accession numbers are provided for each sequence. [file MPP-20-1080-s001.pdf]

```
CBSV_gu563326     -4  IDLQ VVDRPQLSSMNKREEEVTSKIRMGIEAPITFVTGNAQKLKEVKQIF
CBSV_gq329864     -4  IDLQ VVDRPQSLNVAKREEEVTSKFRMGIEAPITFVTGNAQKLKEVKQIF
CBSV_fn434436     -4  IDLQ VVDRPQLLNVAKREEEVTSKFRMGIEAPITFVTGNAQKLKEVKQIF
CBSV_fn434437     -4  IDLQ VIDKPQPSKVAKREEEVTSRIRMGIEAPITFVTGNAQKLKEVKQIF
CBSV_gu563320     -4  IDLQ VIDRPQSSNMTKREEEVTSKIRMGIEAPITFVTGNAQKLKEVKQIF
CBSV_gu563323     -4  IDLQ VVDRPQLSSMTKREEEVTSKVRMGIEAPITFVTGNAQKLKEVKQIF
CBSV_gu563325     -4  IDLQ VVDRSQSTNVAKREEEVTSKIRMGIEAPITFVTGNAQKLKEVKQIF
CBSV_mg570022     -4  IDLQ VVDRSQPSNVAKREEEVTSKIRMGIEAPITFVTGNAQKLKEVKQIF
UCBSV_fj039520    -4  VDTQ TEDLREKEKPELRIESHDGTSRMQMKFPVTFVTGNLGKLAEVRSIL
UCBSV_fj185044    -4  VDTQ TEDLRGREKLELRTESHDRISQLQMKFPVTFVTGNLGKLAEVKSIL
UCBSV_fn433930    -4  VDTQ TKDLRGREKLELRTESHDGTLQMQMKFPVTFVTGNFGKLAEVKSIL
UCBSV_fn433932    -4  VDTQ IKDLRERDEPELRVGSHDGVPRMQMKFPVTFVTGNLGKLAEVKSIL
UCBSV_fn434109    -4  VDTQ K-DLRGGEKPELRTESHDGTPQMQMKFPVTFVTGNFGKLAEVKSIL
UCBSV_hm181930    -4  VDTQ TEDLRGREKLELRTESHDRISQLQMKFPVTFVTGNLGKLAEVKSIL
UCBSV_kx753357    -4  VDTQ TKDLRGREKPELRIESHDGVPQMQMKFPVTFVTGNLGKLAEVKSIL
UCBSV_gq169761    -4  VDTQ TKDLREKEEPELRIESHDGISRMQMKFPVTFVTGNLGKLEEVRSIL

CBSV_gu563326    201  ALSLVRDFLKDSSYFSFAKGVDRDFFIDVQ A
CBSV_gq329864    201  ALSLVRDFLKSSSYFSFAKGLDRDIFIDVQ A
CBSV_fn434436    201  ALSLVRDFLKSSSYFSFAKGLDRDIFIDVQ A
CBSV_fn434437    201  ALSLVRDFLKSSSYFSFAKGLDRDIFIDVQ A
CBSV_gu563320    201  ALSLVRDFLKNSSYFNFAKGVDRDFFIDVQ A
CBSV_gu563323    201  ALSLVRDFLKDSSYFSFAKGVDRDFFIDVQ A
CBSV_gu563325    201  ALSLVRDFLKNSSYFSFAKGVDRDLFIDVQ A
CBSV_mg570022    201  ALSLVRDFLKDSSYFSFAKGVDRDFFIDVQ A
UCBSV_fj039520   201  ALEKVKLFLDNLVVRQEEKRASMALTIDVQ A
UCBSV_fj185044   201  ALEKVKLFLDNLVVKQEKKKAGVALTIDVQ A
UCBSV_fn433930   201  ALEKVKLFLDNLMVKQEKNKASVALTIDVQ A
UCBSV_fn433932   201  ALEKVKLFLDNLVVKQEEKRAKVALTIEVQ A
UCBSV_fn434109   200  ALEKVKSFLDNLVVKQEEKKARVALTIDVQ A
UCBSV_hm181930   201  ALEKVKLFLDNLVVKQEKKKAGVALTIDVQ A
UCBSV_kx753356   201  ALEKVKLYLDNLMVKQEEKKAKVALTIDVQ A
UCBSV_gq169761   201  ALEKVKLFLDNLVVRQEEKRASVALTIDVQ A
```

Figure S1: Relevant sections of a T-coffee alignment of 8 CBSV and 8 UCBSV Ham1 amino acid sequences showing differences in proteolytic cleavage sequences. The junctions where the NIb and CP proteins end are highlighted in yellow. N' Ham1 proteolytic cleavage sequences between the NIb – Ham1 proteins are different in CBSV isolates (green) and UCBSV isolates (pink), indicating potential differences in proteolytic processing. Whereas the C' Ham1 proteolytic cleavage sequence between the Ham1 – CP proteins is conserved in CBSV and UCBSV sequences (blue). The cleave sites where the are shown in yellow Highly conserved regions (>90%) are highlighted in black. Sequences were obtained from the NCBI database; accession numbers are provided for each sequence.
