# Supplementary material for: Cassava brown streak virus Ham1 protein hydrolyses mutagenic nucleotides and is a necrosis determinant
Source: Mol Plant Pathol. 2019 Jun 1;20(8):1080–92. doi: 10.1111/mpp.12813 (PMC6640186; doi:10.1111/mpp.12813)
Supplement: Supplementary file 2 — Fig. S2 Relevant section of T‐coffee alignment of 12 CBSV and 20 UCBSV Ham1 amino acid sequences. The ITPase signature serine‐histidine‐arginine (SHR) motif is highly conserved and was found in all sequences at positions 192–194 (yellow). Highly conserved regions (>90%) are highlighted in black. Sequences were obtained from the NCBI database; accession numbers are provided for each sequence. [file MPP-20-1080-s002.pdf]

```
CBSV_GU563327    181 AEMMAEEKNMISHRFRALSLVRDFLKDSSYFSFAKGVDRDFFIDVQ
CBSV_GQ169758    181 AEMMIEEKNMISHRFRALSLVRDFLKSSSYFSFAKGLDRDIFIDVQ
CBSV_GU563320    181 AEMMAEEKNMISHRFRALSLVRDFLKNSSYFNFAKGVDRDFFIDVQ
CBSV_GU563324    181 AEMMAEEKNMISHRFRALSLVRDFLKDSSYFNFAKGVDRDCFIDVQ
CBSV_N434436     181 AEMMTEEKNMISHRFRALSLVRDFLKSSSYFSFAKGLDRDIFIDVQ
CBSV_GQ329864    181 AEMMTEEKNMISHRFRALSLVRDFLKSSSYFSFAKGLDRDIFIDVQ
CBSV_FN434437    181 AEMMTEEKNMISHRFRALSLVRDFLKSSSYFSFAKGLDRDIFIDVQ
CBSV_GQ169759    181 AEMMTEEKNMISHRFRALSLVRDFLKSSSYFSFAKGLDRDIFIDVQ
CBSV_MG570022    181 AEMMTEEKNMISHRFRALSLVRDFLKDSSYFSFAKGVDRDFFIDVQ
CBSV_LT577537    181 AEMMTEEKNMISHRFRALSLVRDFLKSSSYFSFAKGLDRDIFIDVQ
CBSV_GU563323    181 AEMMAEEKNMISHRFRALSLVRDFLKDSSYFSFAKGVDRDFFIDVQ
CBSV_GU563325    181 AEMMPEEKNILSHRFRALSLVRDFLKNSSYFSFAKGVDRDLFIDVQ
UCBSV_FN433930   181 AEMPSSIKNDFSHRRRALEKVKLFLDNLMVKQEKNKASVALTIDVQ
UCBSV_FN433931   181 AEMPSSIKNDFSHRRRALEKVKLFLDNLMVKQEEKKTRVALTIDVQ
UCBSV_GU205820   181 AEMSSNIKNDFSHRRRALEKVKLFLDNLVVKQEKKKARVALTIDVQ
UCBSV_KX753356   181 AEMSSNIKNDFSHRRKALEKVKLYLDNLMVKQEEKKAKVALTIDVQ
UCBSV_FN433932   181 AEMSSSIKNDFSHRRRALEKVKLFLDNLVVKQEEKRAKVALTIEVQ
UCBSV_FN433933   181 AEMSSSIKNDFSHRRRALEKVKLFLDNLVVKQEEKRAKVALTIEVQ
UCBSV_EU916825   181 AEMPSGIKNEFSHRRRALEKVKLFLDNLVVRQEEKRASMALTIDVQ
UCBSV_EU916826   181 AEMPSGIKNEFSHRRRALEKVKLFLDNQVVRQEKKRASVALTIDVQ
UCBSV_GU205818   181 AEMSSSIKNEFSHRRRALEKVKLYLDNLVVKQEEKKAKVALTIDVQ
UCBSV_GQ169760   181 AEMPSGIKNEFSHRRRALEKVKLFLDNLVVRQEEKRASMALTIDVQ
UCBSV_KR108836   181 AEMPSSIKNDFSHRRRALEKVKLYLDNLVVKQEEKKAKVALTIDVQ
UCBSV_KR108835   181 AEMPSNIKNDFSHRRKALEKVKLYLDNLMVKQEEKKAKVALTIDVQ
UCBSV_FN434109   181 AEMSSSMKNDFSHRRRALEKVKSFLDNLVVKQEEKKARVALTIDVQ
UCBSV_GU205819   181 AEMSSSMKNDFSHRRRALEKVKSFLDNLVVKQEEKKARVALTIDVQ
UCBSV_EU916831   181 AEMSSSIKNDFSHRRRALEKVKLFLDNLVVKQEKKKARVALTIDVQ
UCBSV_EU916832   181 AEMPSSFKNDFSHRRRALEKVKLFLDDLVVKQEKKEARVALTIDVQ
UCBSV_EU916830   181 AEMSSGMKNDFSHRRRALEKVKSFLDNLVVKQEEKKARVALTIDVQ
UCBSV_EU916829   181 AEMSSSMKNDFSHRRRALEKVKSFLDNLVVKQEEKKARVALTIDVQ
UCBSV_EU916827   181 AEMSSSMKNDFSHRRRALEKVKSFLDNLVVKQEEKKARVALTIDVQ
UCBSV_EU916828   181 AEMSSSMKNDFSHRRRALEKVKSFLDNLVVKQEEKKARVALTIDVQ
```

Figure S2: Relevant section of T-coffee alignment of 12 CBSV and 20 UCBSV Ham1 amino acid sequences. The ITPase signature Serine-Histidine-Arginine (SHR) motif is highly conserved and was found in all sequences at positions 192 – 194 (yellow). Highly conserved regions (>90%) are highlighted in black. Sequences were obtained from the NCBI database; accession numbers are provided for each sequence.
