# Supplementary material for: Cassava brown streak virus Ham1 protein hydrolyses mutagenic nucleotides and is a necrosis determinant
Source: Mol Plant Pathol. 2019 Jun 1;20(8):1080–92. doi: 10.1111/mpp.12813 (PMC6640186; doi:10.1111/mpp.12813)
Supplement: Supplementary file 3 — Fig. S3 SDS‐PAGE of purified CBSV_Tanza (gel A) and UCBSV_Kikombe (gel B) Ham1 proteins (25 kDa). Lanes in gel A correspond to separate fractions B4–B13 that were eluted from the AKTA machine during protein purification at a range of imidazole concentrations. Lane D in gel B refers to the UCBSV Ham1 protein which had been dialysed into the storage buffer. To prepare the protein samples for loading, 10 μL of loading buffer (4% SDS, 0.25 M Tris.HCl pH 6.8, 20% glycerol, 0.004% bromophenol blue, 10% β‐mercaptoethanol), 1 μL of protein sample, and 9 μL of water were mixed and heated at 95 °C for 5 min. A TruPAGE Precast Gel (Sigma Aldrich) was loaded with 10 μL of each prepared sample and 10 μL of PageRuler Protein Ladder (Thermo Fisher Scientific). The gel was run at 220 V for 40 min. The gel was stained with 20 mL InstantBlue Protein Stain (Sigma Aldrich) and analysed under white light using the ChemDoc Bio Rad System. Images were taken using the Quantity One 1D software (Bio‐Rad). [file MPP-20-1080-s003.pdf]

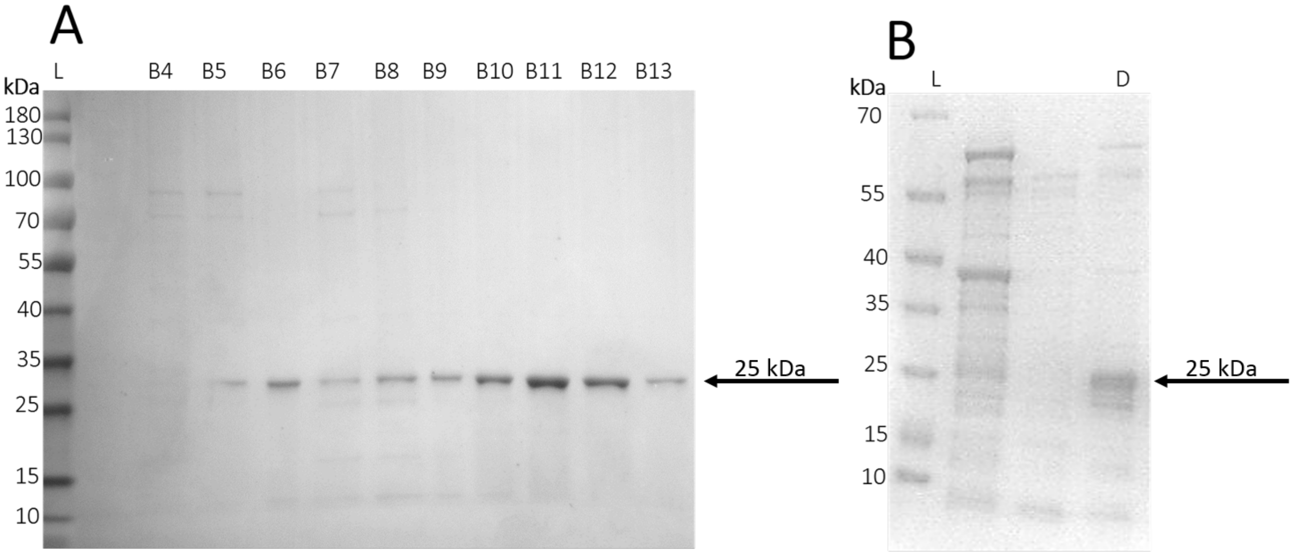

Figure S3: SDS-PAGE of purified CBSV_Tanza (gel A) and UCBSV_Kikombe (gel B) Ham1 proteins (25 kDa). Lanes in gel A correspond separate fractions B4 – B13 that were eluted from the AKTA machine during protein purification at a range of imidazole concentrations. Lane D in gel B refers to UCBSV Ham1 protein which had been dialysed into the storage buffer. To prepare the protein samples for loading, 10 µL of loading buffer (4% SDS, 0.25 M Tris-HCl - pH 6.8, 20% glycerol, 0.004% bromophenol blue, 10% β-mercaptoethanol), 1 µL of protein sample and 9 µL of water were mixed and heated at 95ºC for 5 mins. A TruPAGE Precast Gel (Sigma Aldrich) was loaded with 10 µL of each prepared sample and 10 µL of PageRuler Protein Ladder (Thermo Fisher Scientific). The gel was run at 220V for 40 mins. The gel was stained with 20 ml InstantBlue Protein Stain (Sigma Aldrich) and analysed under white light using the ChemDoc Bio-Rad System and images were taken using the Quantity One 1D software (Bio-Rad).
