# Supplementary material for: Cassava brown streak virus Ham1 protein hydrolyses mutagenic nucleotides and is a necrosis determinant
Source: Mol Plant Pathol. 2019 Jun 1;20(8):1080–92. doi: 10.1111/mpp.12813 (PMC6640186; doi:10.1111/mpp.12813)
Supplement: Supplementary file 4 — Fig. S4 Enzyme assay results from the heat inactivation experiment to test for loss of CBSV_Tanza Ham1 ITPase activity. Incubation of 0.2 mM dITP with active CBSV_Tanza Ham1 protein (1.3 μg) resulted in a phosphate concentration of 136 μM. Heating the CBSV_Tanza Ham1 at 95 °C for 10 min to 1 h resulted in a 41–43% reduction in phosphate concentration, indicating inactivation of its pyrophosphohydrolase activity. Control assays were set where BSA protein (1.3 μg) was added, which produced a comparable phosphate concentration to assays where CBSV_Tanza Ham1 had been heat inactivated, indicating that BSA could be used as a control for addition of protein to assay samples. Low background phosphate concentrations were found in negative controls: (1) containing 0.2 mM dITP in reaction buffer with the addition of 0.1 units of yeast inorganic pyrophosphatase, (2) 0.2 mM dITP in reaction buffer, and (3) water. [file MPP-20-1080-s004.pdf]

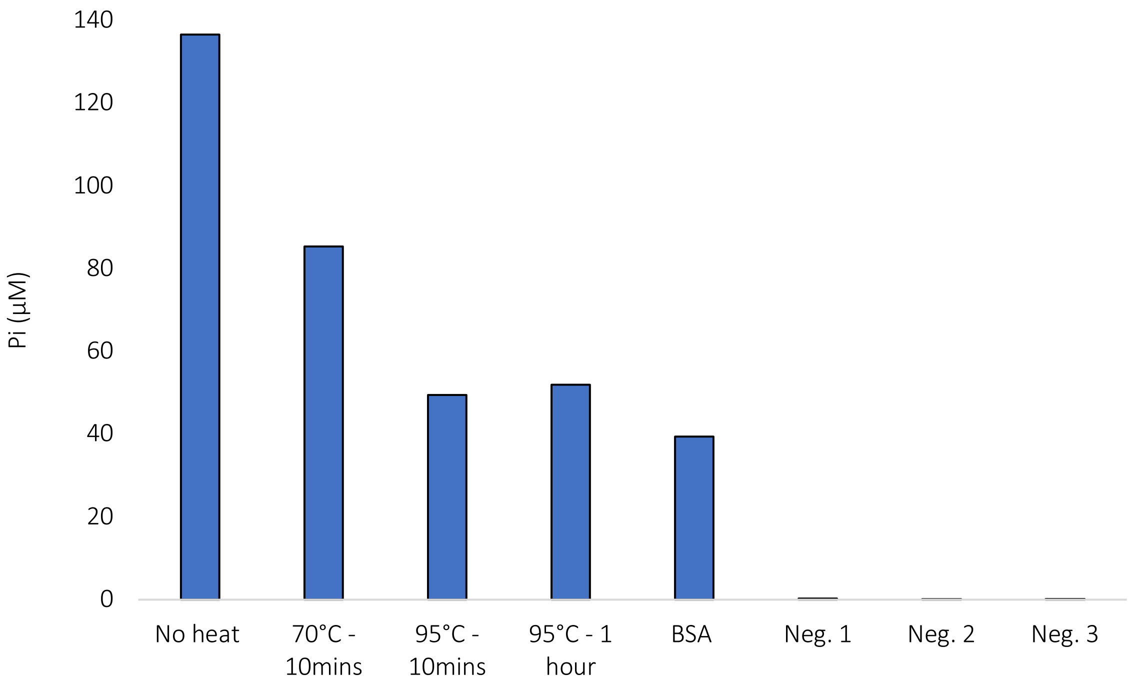

Figure S4: Enzyme assay results from the heat inactivation experiment to test for loss of CBSV Tanza Ham1 ITPase activity. Incubation of 0.2 mM dITP with active CBSV Tanza Ham1 protein (1.3 µg) resulted in a phosphate concentration of 136 µM. Heating the CBSV Tanza Ham1 at 95°C for 10 mins – 1 hour resulted in a 41 – 43% reduction in phosphate concentration, indicating inactivation of its pyrophosphohydrolase activity. Control assays were set where BSA protein (1.3 µg) was added, which produced a comparable phosphate concentration to assays where CBSV Tanza Ham1 had been heat inactivated, indicating that BSA could be used as a control for addition of protein to assay samples. Low background phosphate concentrations were found in negative controls: 1) containing 0.2 mM dITP in reaction buffer with the addition of 0.1 units of yeast inorganic pyrophosphatase, 2) 0.2 mM dITP in reaction buffer and 3) water.
