# Supplementary material for: Cassava brown streak virus Ham1 protein hydrolyses mutagenic nucleotides and is a necrosis determinant
Source: Mol Plant Pathol. 2019 Jun 1;20(8):1080–92. doi: 10.1111/mpp.12813 (PMC6640186; doi:10.1111/mpp.12813)
Supplement: Supplementary file 5 — Fig. S5 5‐FU resistance agar plate growth assays. The wild‐type yeast strain BY4742 was transformed with pYES2 plasmids containing Ham1 sequences from CBSV_Nampula, CBSV_Tanza, UCBSV_Kikombe and yeast. Transformant yeast was cultured and plated onto SD media as ten‐fold serial dilutions onto test plates containing 2% galactose and 10 µg/mL 5‐FU or control plates containing 2% galactose only. Colony growth was imaged after 72 h. Results were consistent in three separate experiments. [file MPP-20-1080-s005.pdf]

	Control (-) 5-FU	Test (+) 5-FU
pYES2	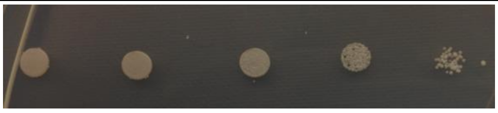	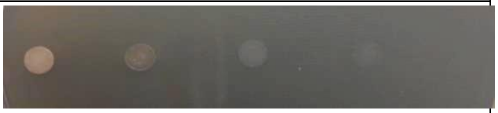
pYES2-HAM1 (CBSV-Nampula)	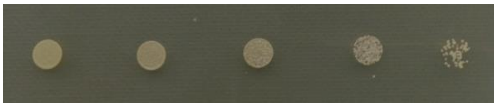	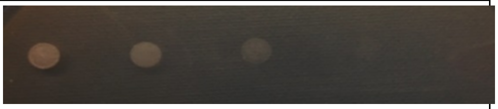
pYES2-HAM1 (CBSV-Tanza)	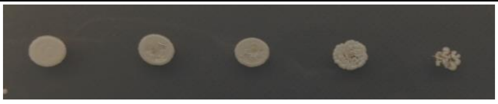	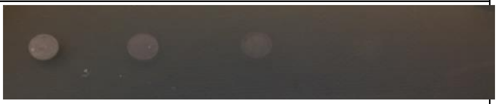
pYES2-HAM1 (UCBSV-Kikombe)	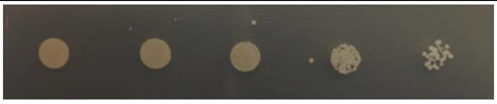	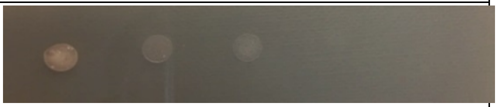
pYES2-HAM1 (*S. cerevisiae*)	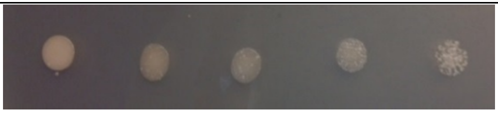	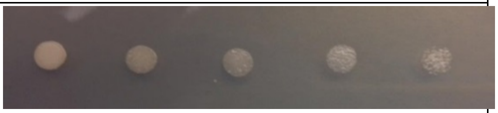
	10^{-1} 10^{-2} 10^{-3} 10^{-4} 10^{-5}	10^{-1} 10^{-2} 10^{-3} 10^{-4} 10^{-5}

S5: 5-FU resistance agar plate growth assays. The wild-type yeast strain BY4742 was transformed with pYES2 plasmids containing Ham1 sequences from CBSV Nampula, CBSV Tanza, UCBSV Kikombe and yeast. Transformant yeast were cultured and plated onto SD media as ten-fold serial dilutions onto test plates containing 2% galactose and 10 µg/ml 5-FU or control plates containing 2% galactose only. Colony growth was imaged after 72 hours. Results were consistent in three separate experiments.
