# Supplementary material for: Cassava brown streak virus Ham1 protein hydrolyses mutagenic nucleotides and is a necrosis determinant
Source: Mol Plant Pathol. 2019 Jun 1;20(8):1080–92. doi: 10.1111/mpp.12813 (PMC6640186; doi:10.1111/mpp.12813)
Supplement: Supplementary file 6 — Fig. S6 5‐FU resistance liquid growth assays. The wild‐type yeast strain BY4742 was transformed with pYES2 plasmids containing Ham1 sequences from CBSV_Nampula, CBSV_Tanza, UCBSV_Kikombe and yeast. Transformant yeast was cultured in liquid SD media containing 2 galactose and 10 µg/mL 5‐FU. The cell density (OD600) was taken at 4, 8, 12, 24, 48 and 72 h. Yeast transformed with yeast Ham1 sequence demonstrated relatively high levels of growth. Yeast transformed with U/CBSV Ham1 sequences showed low levels of growth, comparable to the negative control (empty pYES2 plasmid). This indicates that unlike the yeast Ham1 sequence, the U/CBSV Ham1 sequences were unable to protect against mutagenic 5‐FU. Each result is the mean OD600 value from three replicate samples (n = 3) ± SE. Results were consistent in three separate experiments. [file MPP-20-1080-s006.pdf]

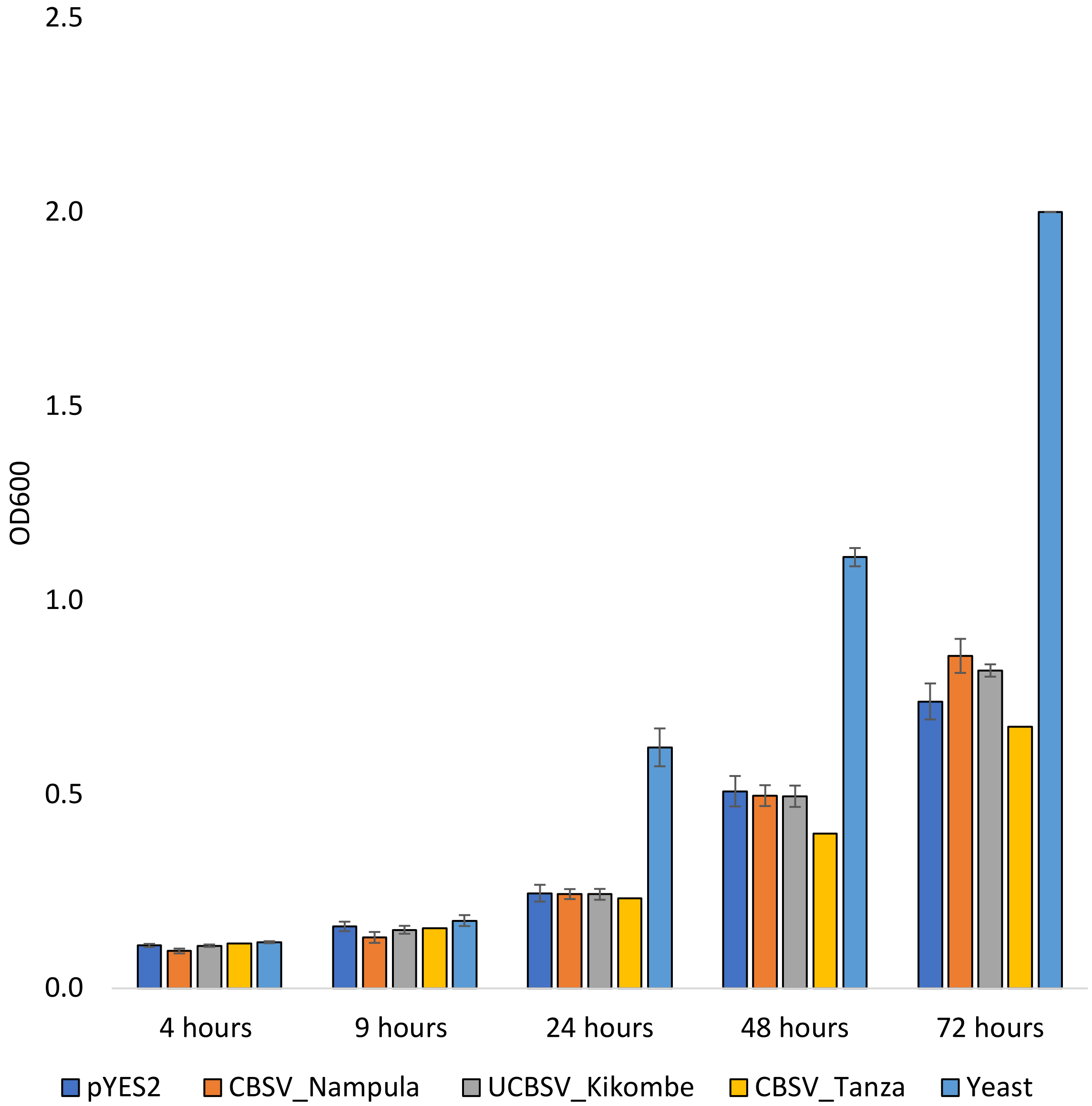

S6: 5-FU resistance liquid growth assays. The wild-type yeast strain BY4742 was transformed with pYES2 plasmids containing Ham1 sequences from CBSV Nampula, CBSV Tanza, UCBSV Kikombe and yeast. Transformant yeast were cultured liquid SD media containing 2% galactose and 10 µg/ml 5-FU. The cell density (OD_{600}) was taken at 4, 8, 12, 24, 48 and 72 hours. Yeast transformed with yeast Ham1 sequence demonstrated relatively high levels of growth. Whereas as yeast transformed with U/CBSV Ham1 sequences showed low levels of growth, comparable to the negative control (empty pYES2 plasmid). This indicates that unlike the yeast Ham1 sequence, the U/CBSV Ham1 sequences were unable to protect against mutagenic 5-FU. Each result is the mean OD_{600} value from three replicate samples (n = 3) ± S.E. Results were consistent in three separate experiments.
