# Supplementary material for: Cassava brown streak virus Ham1 protein hydrolyses mutagenic nucleotides and is a necrosis determinant
Source: Mol Plant Pathol. 2019 Jun 1;20(8):1080–92. doi: 10.1111/mpp.12813 (PMC6640186; doi:10.1111/mpp.12813)
Supplement: Supplementary file 7 — Fig. S7 The replacement of CBSV_Tanza Ham1 sequence (blue) with UCBSV Ham1 sequence (red) in the CBSV_UHam1 IC. To ensure proteolytic cleavage of UCBSV Ham1 sequence from the CBSV Tanza polyprotein the NIb‐Ham1 protease cleavage sequence IDLQV was maintained at the start of the UCBSV Ham1 sequence TKD. [file MPP-20-1080-s007.pdf]

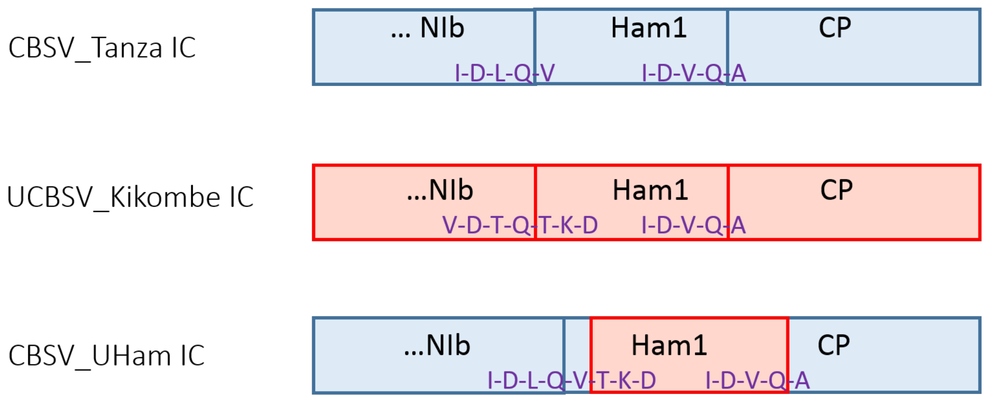

Figure S7: Schematic showing the replacement of CBSV Tanza Ham1 sequence (blue) with UCBSV Ham1 sequence (red) in the CBSV_UHam1 IC. To ensure proteolytic cleavage of UCBSV Ham1 sequence from the CBSV Tanza polyprotein the NIb – Ham1 protease cleavage sequence I-D-L-Q-V was maintained at the start of the UCBSV Ham1 sequence: T-K-D.
