# Supplementary material for: Cassava brown streak virus Ham1 protein hydrolyses mutagenic nucleotides and is a necrosis determinant
Source: Mol Plant Pathol. 2019 Jun 1;20(8):1080–92. doi: 10.1111/mpp.12813 (PMC6640186; doi:10.1111/mpp.12813)
Supplement: Supplementary file 8 — Table S1 Games–Howell one‐way ANOVA tests to compare mean phosphate concentration in enzyme assay reactions with CBSV_Tanza Ham1 incubated with the non‐canonical nucleotides XTP and dITP, and a range of canonical nucleotides. [file MPP-20-1080-s008.pdf]

Table S1: Games-Howell one-way ANOVA tests to compare mean phosphate concentration in enzyme assay reactions with CBSV Tanza Ham1 incubated with the non-canonical nucleotides XTP and dITP and a range of canonical nucleotides.

**Protein**			**CBSV Ham1**	
		Non-canonical nucleotides	XTP	dITP
Canonical nucleotides		Mean Pi (µM)	198	172
	Mean Pi (µM)			
dGTP	134	Difference (µM)	64	38
		Sig. p value	0.828	0.979
GTP	90	Difference (µM)	108	82
		Sig. p value	0.062	0.139
UTP	48	Difference (µM)	150	124
		Sig. p value	0 .030 *	0.040 *
dTTP	27	Difference (µM)	171	145
		Sig. p value	0.008 **	0.011 *
dATP	19	Difference (µM)	179	152
		Sig. p value	0.016 *	0.019 *
dCTP	12	Difference (µM)	186	160
		Sig. p value	0.009 **	0.011 *
CTP	13	Difference (µM)	14	158
		Sig. p value	0.013 *	0.016 *
ATP	9	Difference (µM)	189	163
		Sig. p value	0.019 *	0.023 *
