# Supplementary material for: Cassava brown streak virus Ham1 protein hydrolyses mutagenic nucleotides and is a necrosis determinant
Source: Mol Plant Pathol. 2019 Jun 1;20(8):1080–92. doi: 10.1111/mpp.12813 (PMC6640186; doi:10.1111/mpp.12813)
Supplement: Supplementary file 9 — Table S2 Games–Howell one‐way ANOVA tests to compare mean phosphate concentration in enzyme assay reactions with UCBSV_Kikombe Ham1 incubated with the non‐canonical nucleotides XTP and dITP, and a range of canonical nucleotides. [file MPP-20-1080-s009.pdf]

Table S2: Games-Howell one-way ANOVA tests to compare mean phosphate concentration in enzyme assay reactions with UCBSV Kikombe Ham1 incubated with the non-canonical nucleotides XTP and dITP and a range of canonical nucleotides.

| Protein | | | UCBSV Ham1 | |
|---|---|---|---|---|
| | | Non-canonical nucleotides | XTP | dITP |
| Canonical nucleotides | | Mean Pi (µM) | 190 | 178 |
| | Mean Pi (µM) | | | |
| dGTP | 100 | Difference (µM) | 90 | 79 |
| | | Sig. p value | 0.180 | 0.227 |
| GTP | 83 | Difference (µM) | 106 | 95 |
| | | Sig. p value | 0.095 | 0.107 |
| UTP | 59 | Difference (µM) | 131 | 119 |
| | | Sig. p value | 0.074 | 0.073 |
| dTTP | 20 | Difference (µM) | 70 | 157 |
| | | Sig. p value | 0.051 * | 0.049 * |
| dATP | 24 | Difference (µM) | 165 | 154 |
| | | Sig. p value | 0.052 * | 0.053 * |
| dCTP | 26 | Difference (µM) | 164 | 153 |
| | | Sig. p value | 0.054 * | 0.052 * |
| CTP | 32 | Difference (µM) | 158 | 147 |
| | | Sig. p value | 0.063 | 0.062 |
| ATP | 9 | Difference (µM) | 180 | 169 |
| | | Sig. p value | 0.050 * | 0.048 * |
