# Supplementary material for: Cassava brown streak virus Ham1 protein hydrolyses mutagenic nucleotides and is a necrosis determinant
Source: Mol Plant Pathol. 2019 Jun 1;20(8):1080–92. doi: 10.1111/mpp.12813 (PMC6640186; doi:10.1111/mpp.12813)
Supplement: Supplementary file 10 — Table S3 Primers used to amplify CBSV and UCBSV Ham1 sequences during cloning into the POPINF vector. Sequences overlapping with the POPINF vector are shown in red. [file MPP-20-1080-s010.pdf]

Table S3: Primers used to amplify CBSV and UCBSV Ham1 sequences during cloning into the POPINF vector. Sequences overlapping with the POPINF vector are shown in red.

Primer name	Sequence 5'3
POPINF_CBSV_Ham1_Fw	AAGTTCTGTTTCAGGGCCCGGTGGTGGACAGGTCTCAGCC
POPINF_CBSV_Ham1_Rv	ATGGTCTAGAAAGCTTTAGCTTGAACATCAATAAAGAAATCACG
POPINF_UCBSV_Ham1_Fw	AAGTTCTGTTTCAGGGCCCGACAAAGGATTTGAGAGGAAGAGAG
POPINF_UCBSV_Ham1_Rv	ATGGTCTAGAAAGCTTTACTGCACATCAATTGTTAGAGCCAC
