# Supplementary material for: Cassava brown streak virus Ham1 protein hydrolyses mutagenic nucleotides and is a necrosis determinant
Source: Mol Plant Pathol. 2019 Jun 1;20(8):1080–92. doi: 10.1111/mpp.12813 (PMC6640186; doi:10.1111/mpp.12813)
Supplement: Supplementary file 11 — Table S4 Primers used to amplify PCR fragments to construct the CBSV_mutHam and CBSV_UHam ICs. The nucleotide sequence encoding the SHR to SAA mutation is shown in blue. The nucleotide sequence encoding the UCBSV Ham1 is shown in red. [file MPP-20-1080-s011.pdf]

Table S4: Primers used to amplify PCR fragments to construct the CBSV_mutHam and CBSV_UHam ICs. The nucleotide sequence encoding the SHR to SAA mutation are shown in blue. The nucleotide sequence encoding the UCBSV Ham1 are shown in red.

Infectious clone	Primer	Sequence 5' – 3'	Size (bp)	Target
CBSV_mutHam	SHR_mut_F1_Fw	GAAATATAATGAACCTGTTCGAGTGGGGTTGGTAAAC	1916	NIb – Ham1
	SHR_mut_F1_Rv	TCCTTCAAAAAGTCTCTCACTAATGACAGAGCCCGAAAGGCAGCAGATATCATATTCTTC		
	SHR_mut_F2_Fw	GAAGAATATGATATCTGCTGCCTTTCGGGCTCTGTCATTAGTGAGAGACTTTTTGAAGGA	1810	Ham1 – 3'UTR
	SHR_mut_F2_Rv	GGCTGGCTGGTGGCAGGATATATTGTGGTGTAAAC		
CBSV_UHam	UCBSV_HF1_Fw	GAAATATAATGAACCTGTTCGAGTGGGGTTGGTAAAC	1336	CBSV NIb
	UCBSV_HF1_Rv	CAGGCTTCTCTCTTCCTCTCAAATCCTTTGTGTCCACCACTTGTAAGTCAATGTAACAAT		
	UCBSV_HF2_Fw	ACAAAGGATTTGAGAGGAAGAGAGAAGCCTGAGTTGAGAATTGAGAGCCAT	677	UCBSV Ham1
	UCBSV_HF2_Rv	CTGCACATCAATTGTTAGAGCCACCTTTGCCTTCTTCTCCTCTTGTTTCACCATC		
	UCBSV_HF3_Fw	GAAGGCAAAGGTGGCTCTAACAATTGATGTGCAGGCAATTGACAAGGATGAGATTGA	1729	CBSV CP – 3'UTR
	UCBSV_HF3_Rv	GGCTGGCTGGTGGCAGGATATATTGTGGTGTAAAC		
