# Supplementary material for: Cassava brown streak virus Ham1 protein hydrolyses mutagenic nucleotides and is a necrosis determinant
Source: Mol Plant Pathol. 2019 Jun 1;20(8):1080–92. doi: 10.1111/mpp.12813 (PMC6640186; doi:10.1111/mpp.12813)
Supplement: Supplementary file 12 — Table S5 Relative expression of the CBSV_Nampula Ham1 gene in three transgenic Nicotiana tabacum lines compared with wild‐type; detected by qPCR as described in the Experimental Procedures section. [file MPP-20-1080-s012.pdf]

Table S5: Relative expression of the CBSV Nampula Ham1 gene in three transgenic *Nicotiana tabacum* lines compared with wild-type; detected by qPCR as described in the 'Experimental Procedures'.

Nicotiana tabacum line	Relative CBSV Nampula Ham1 expression
Ham 1.1	23,170
Ham 1.2	6,608
Ham 1.3	50,360
Wild-type	1
